# The effect of celery (*Apium graveolens*) powder on cardiometabolic factors in overweight/obese individuals with type 2 diabetes mellitus: A pilot randomized, double‐blinded, placebo‐controlled clinical trial

**DOI:** 10.1002/fsn3.3493

**Published:** 2023-06-08

**Authors:** Mohammad Ali Mohsenpour, Mahsa Samadani, Zeinab Shahsavani, Mohammad Taghi Golmakani, Gholam Reza Pishdad, Maryam Ekramzadeh

**Affiliations:** ^1^ Nutrition Research Center, Department of Clinical Nutrition, School of Nutrition and Food Sciences Shiraz University of Medical Sciences Shiraz Iran; ^2^ Student Research Committee Shiraz University of Medical Sciences Shiraz Iran; ^3^ Department of Nutrition, School of Allied Medical Sciences Ahvaz Jundishapur University of Medical Sciences Ahvaz Iran; ^4^ Department of Community Nutrition, School of Nutrition and Food Sciences Shiraz University of Medical Sciences Shiraz Iran; ^5^ Department of Food Science and Technology, School of Agriculture Shiraz University Shiraz Iran; ^6^ Endocrine and Metabolism Research Center Shiraz University of Medical Sciences Shiraz Iran

**Keywords:** *Apium graveolens*, celery, hyperglycemia, hyperlipidemia, hypertension, weight loss

## Abstract

Celery (*Apium graveolens*) was shown to have beneficial effects on cardiometabolic factors in animal models. As the progression of type 2 diabetes mellitus (T2DM) adversely affects cardiometabolic factors, we aimed to assess the effects of celery powder on glycemic and anthropometric indices, lipid profile, liver function, oxidative stress, and blood pressure of individuals with T2DM. In a pilot randomized, double‐blinded, placebo‐controlled clinical trial, 50 eligible adults with T2DM were randomly divided into two groups of intervention and control to consume either 750 mg of celery powder (obtained from fresh celery) or placebo along with a low‐calorie diet for 12 weeks, respectively. Dietary intake, physical activity, and cardiometabolic factors were assessed before and at the end of the study. Thirty‐six patients finished the study (18 in each group). Consumption of celery powder significantly reduced body fat percentage (*p* = .021). Between‐group analysis for changes in cardiometabolic factors did not show significant differences. Although malondialdehyde was reduced in the intervention group and increased in the control group, between‐group changes were not significant. Although the insulin‐level change was statistically insignificant, a clinical improvement was observed in the intervention group. A 750‐mg daily supplementation of celery powder for 12 weeks did not improve the cardiometabolic factors of patients with T2DM. Further studies are suggested.

## Graphical abstract

The prevalence of type 2 diabetes mellitus is rapidly increasing. In order to alleviate its complications, the celery plant is proposed to be effective. Thus, in this study, the effect of celery powder on metabolic factors in patients with type 2 diabetes was investigated.

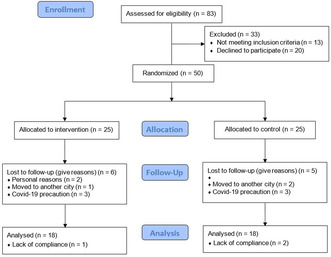

## 1 INTRODUCTION

Celery (*Apium graveolens*), a member of the Apiaceae family, grows in high‐moisture, low‐temperature climates (Kooti & Daraei, 2017). It is a biennial plant that grows to 100 cm, and its different parts including leaves, roots, and stalks have various health‐promoting compounds (Hedayati et al., 2019). Apigenin, hesperidin, luteolin, quercetin, and rosmarinic acid are flavonoids found in celery (Priecina & Karklina, 2014). Of minerals, sodium, potassium, magnesium, calcium, and vitamins such as beta carotene and vitamin C are available in celery. In addition, this herb contains a high amount of fiber (Fazal & Singla, 2012).

In traditional medicine, different parts of celery are used to improve liver function. Also, some preventive characteristics for cardiovascular diseases (CVD), hypertension, and high serum glucose have been found for celery (Khalid et al., 2016). Its roots seem to balance calcium and potassium levels in heart tissues and chemical compounds in its leaves can scavenge harmful radicals. Furthermore, roots and leaves empower enzyme activities by restoration of liver glutathione, glutathione peroxidase, catalase, and regulating lipid peroxidation (Kooti et al., 2015). Celery is also used in medical nutrition therapy and weight management diets (Sowbhagya, 2014).

Obesity has been shown to increase the risk and severity of type 2 diabetes mellitus (T2DM) (Carbone et al., 2019). As a result, individuals with T2DM face adverse changes in cardiometabolic factors including increases in weight, hyperlipidemia, and hemoglobin A1c (HbA1c). As T2DM progresses, impaired glycemic control intensifies (Steinarsson et al., 2018). Consequently, in line with hyperglycemia, proinflammatory markers and oxidative stress increase. This is while these factors have shown bidirectional relation with insulin resistance which in turn can exacerbate poor glycemic control (Oguntibeju, 2019). In addition, uncontrolled blood glucose leads to increased serum levels of alanine transaminase (ALT) and aspartate aminotransferase (AST), as indicators of liver function (Shibabaw et al., 2019). Eventually, T2DM by means of the abovementioned biochemical imbalance can increase the risk of all types of cancers (Rey‐Reñones et al., 2021), hepatic diseases (Targher et al., 2018), and mortality (Carrillo‐Larco et al., 2019).

It has been found that celery leaves could have a role in decreasing preprandial and postprandial serum glucose levels, but no effect has been shown on insulin levels (Yusni et al., 2018). Based on the results of animal studies, active compounds in celery such as luteolin and apigenin are proposed to improve hyperglycemia. Furthermore, studies on animal models revealed antihyperlipidemic (triglycerides [TG], low‐density lipoprotein [LDL‐c], and high‐density lipoprotein [HDL‐c]), antiobesity, and antihypertensive characteristics of celery extract (Hedayati et al., 2019). The hypotensive effect of celery seed (Gharouni & Sarkarati, 2000; Shayani Rad et al., 2022) and the extract was also shown in the human model (Baradaran et al., 2014; Dewi et al., 2010; Madhavi et al., 2013).

Despite the various health‐promoting compounds that exist in the celery herb (*A. graveolens*), only a few studies have been conducted to investigate its effects in the context of controlled clinical trials. Moreover, the published investigations assessed the health‐promoting effect of celery seed (Gharouni & Sarkarati, 2000; Shayani Rad et al., 2022) or extract (Baradaran et al., 2014; Dewi et al., 2010; Madhavi et al., 2013), while no studies have investigated the whole plant. Thus, we aimed to study the effect of celery powder on cardiometabolic factors including glycemic and anthropometric status, lipid profile, liver function, oxidative stress, and blood pressure of overweight/obese individuals with T2DM.

## 2 MATERIALS AND METHODS

### 2.1 Study design and participants

The present pilot‐parallel randomized, double‐blinded, placebo‐controlled clinical trial is aimed to investigate the effect of celery powder on glycemic, metabolic, and anthropometric indices in individuals suffering from T2DM aged 20–60 years who are overweight or obese. The study was carried out from February 2021 until July 2021.

Subjects with the following characteristics were eligible to be recruited in the study: (1) individuals of both genders aged between 20 and 60 years old, (2) having BMI between 25 and 40 (kg/m^2^), (3) with controlled T2DM (HbA1c <7%; Nathan & DCCT/EDIC Research Group, 2014), (4) patients with hyperlipidemia on medication, (5) not having taken weight loss medication for 3 months prior to the study, (6) not having any history of thyroid, renal, hepatic, cardiovascular, metabolic (except for T2DM), endocrine, or congenital disorders, and (7) not taking antioxidant supplements including vitamin E, vitamin C, lipoic acid, and omega‐3 fatty acids. Moreover, participants were excluded from the study in case of having a history of slimming or gastric bypass surgery, being pregnant or in the lactating period, or having tested positive for COVID‐19.

Patient enrollment was undertaken at Endocrine Diseases Clinic, Shiraz, Iran. All volunteers were informed about the study aims and procedures, and their rights before inclusion into the study. A written consent form was signed by all participants who were willing to participate in the study. The study protocol was in accordance with the Declaration of Helsinki and approved by the ethics committee of Shiraz University of Medical Sciences (SUMS) with the reference number of IR.SUMS.REC.1398.1381 and registered in the Iranian Registry of Clinical Trials (IRCT.ir; registration ID: IRCT20100223003408N8; registration date: March 1, 2021).

The block randomization (1:1 ratio) was used to randomly assign 50 participants to either the intervention or control group. The random sequence was kept in sealed opaque envelopes and later revealed to investigators and participants. The randomization and concealing sequences were done by an individual not involved in the study.

Patients were screened for eligibility, informed consent was obtained, randomly allocated to the two groups, and demographic data were recorded by the investigator. Anthropometric and blood pressure measurements were also undertaken. In addition, dietary intake and physical activity were also assessed. Participants also received a referral note for a blood test at the beginning and after completion of the study to measure fasting blood sugar (FBS), insulin, lipid profile, liver enzymes, and malondialdehyde (MDA).

By using adjusted ideal body weight, the total energy expenditure (TEE) for participants was calculated by a standard formula. A low‐calorie diabetic dietary plan was designed for each patient with a reduction of 500 kcal/day with the macronutrient composition of 50%–55%, 15%–20%, and 30%–35% from carbohydrate, protein, and fat, respectively. Participants were educated on how to adhere to the prescribed dietary plan.

The allocation envelope was then opened to disclose the allocated group named A or B. Thus, the participants entered a 12‐week study based on the allocated group. During the study period, participants consumed three capsules per day each containing 250 mg celery powder or medicinal starch powder (750 mg/day, based on the previous studies; Yusni et al., 2018) along with the hypocaloric diabetic dietary plan in the intervention or control group, respectively. Capsules were named A or B in regard to the study groups and were similar in appearance, size, and color to mask both participants and investigators.

Participants were asked not to change their physical activity level or take any nutritional supplements. During the study, participants received weekly telephone calls for checking adherence to the study protocol and possible side effects. Moreover, participants were followed up with regard to any possible COVID‐19 infection. In case of a positive infection, participants were excluded from the study.

### 2.2 Intervention

Celery powders were prepared prior to the trial running. Fresh celery was purchased from markets in Shiraz, washed, and disinfected. Then the whole celery, including stems and leaves, were chopped. Chopped celeries dispersed on the tray with a thin layer and placed in the cabin dryer (Proctor & Schwartz) at 38°C, periodically weighted till constant weight was obtained (24–48 h). Thus, 41.91% of the weight was reduced due to the evaporation of the water content of celery. Afterward, dried celery was grounded to achieve uniform powder. Samples of the powders were collected in order to test for magnesium (atomic absorption spectroscopy), potassium (atomic absorption spectroscopy), and fiber content (enzymatic‐gravimetric method). The permissions for the collection of celery were obtained. The collecting of celery complied with relevant institutional, national, and international guidelines and legislation, the IUCN Policy Statement on Research Involving Species at Risk of Extinction, and the Convention on the Trade in Endangered Species of Wild Fauna and Flora.

The antioxidant activity of the powder was measured by the DPPH radical (DPPH°) method developed by Xu and Chen (2013). In this method, the antioxidant activity or the radical scavenging activity of the sample is estimated by the ability of the antioxidant to donate hydrogen which affects DPPH°. Mixing the substrate with DPPH° solution (2,2‐diphenyl‐1‐picrylhydrazyl radical) causes the reduction. Consequently, the reagent color will change from violet to pale yellow (Mazidi et al., 2012). Finally, the sample concentration which was able to provide 50% inhibition (IC50) was determined (Farahmand et al., 2017). All these procedures were done under the supervision of MTG in the pilot plant and laboratory of the Department of Food Sciences and Technology, College of Agriculture, Shiraz University, Shiraz, Iran. The chemical characteristics of the celery powder are stated in Table 1.

**TABLE 1:** Celery powder characteristic.

Property	Result
Fiber (g/100 g celery powder)	10.51
Potassium (g/100 g celery powder)	2.7
Magnesium (g/100 g celery powder)	0.3
IC50 (mg/mL)	0.53

*Note*: RDA for magnesium: 400–420 and 310–320 mg daily for 19‐ to 51‐year‐old men and women, respectively.

Abbreviations: IC50, half‐maximal inhibitory concentration; RDA, recommended daily allowance.

Then, celery powder (obtained from stem and leaves) and the starch were separately stirred to achieve uniform powders. To sterilize, a thin layer of each powder (celery or starch) was radiated under a 359‐nm UV lamp for 1 h. The sterilized powders were filled in 250 mg capsules, which are the same in appearance, in the Pharmacy Faculty, Shiraz University of Medical Sciences, Shiraz, Iran.

### 2.3 Dietary intake measurement

Weighted food records for 3 days (two weekdays and a weekend) were recorded to assess total energy, macronutrient, and micronutrient intake at the beginning and at the end of the study. Participants were trained to record the food items and drinks based on the household measurement scales, time of consumption, and methods of preparations by the investigation team. Reported food intakes were converted to grams per day based on usual household measures of Iranian and analyzed using Nutritionist IV software (First Databank, San Bruno, CA, USA) modified for Iranian food by a trained nutritionist.

### 2.4 Physical activity measurement

Participants reported their physical activity using the MET questionnaire for 3 days (two weekdays and a weekend) at the initiation and at the completion of the study. The metabolic equivalent per hour coefficient of each reported physical activity was multiplied by the duration of the activity (hour) to calculate the MET/day for each participant.

### 2.5 Anthropometric measurement

Anthropometrics were measured using standard procedures. Height was measured using a stadiometer attached to the wall to the nearest 0.1 cm, while the participant was standing with no shoes or hat, looking forward, heels, buttocks, shoulders, and the back of the head were touching the wall. Waist and hip circumference (HC) were recorded using tape measures with an accuracy of 0.1 cm just above the hip bone and the largest part around the hip, respectively. Participants stood in the middle of a body analyzer (OMRON, model: BF510) with the least possible clothing to measure body weight and body fat percent. BMI was also calculated using the standard formula: weight (kg)/height^2^ (m). All the assessments were done three times by the same assessor to ensure maximum accuracy.

### 2.6 Blood marker and blood pressure measurements

Blood pressure was checked at the baseline and during the final visit using the standard procedure, after 15 min of resting in a sitting position (sphygmomanometer, mercury, ALPK1, Japan). Blood pressure measurement were repeated three times with 15 min intervals for more accuracy. The final blood pressure report was the average acquired from these assessments. The laboratory analyzed samples for FBS, lipid profile including triglycerides, LDL, HDL, and total cholesterol, and ALT and AST using an autoanalyzer (BT‐1500 Biotenica). Fasting insulin level was assessed using enzyme‐linked immunoassay kits (Monobind, Inc.). Serum concentrations of MDA were measured by the spectrophotometric method with a modified thiobarbituric acid method (intra‐assay coefficients = 5.5%, interassay coefficients = 5.9%). Laboratory test results were sent to the investigation team by the laboratory management, anonymous, and coded based on the referral notes. Homeostatic model assessment for insulin resistance (HOMA‐IR) was calculated as [(FBS [mg/dL] × insulin [mIU/mL])/405] (Abdollahi et al., 2019). Homeostatic model assessment for β‐cell function (HOMA‐B) was also calculated by standard formula as [(360 × insulin [mIU/mL])/(FBS [mg/dL] − 63)] (Abdollahi et al., 2019).

### 2.7 Statistical analysis

Based on the previous investigation (Yusni et al., 2018), considered fasting blood glucose as the primary outcome, 5% error, and 90% power, the minimum required sample size was calculated as 22 for each group. Thus, with a possible attrition rate of 10%, 25 adults with T2DM would have been included in each group.

Demographic data were summarized as number and percent for categorical variables, and median and interquartile ranges (25th and 75th percentiles) for non‐normal continuous variables. Data were analyzed for normality. For skewed data, the Mann–Whitney *U* test and Wilcoxon signed rank test were used for between‐group and within‐group comparisons, respectively, and median and interquartile ranges (25th and 75th percentiles) were used for summarizing the results. In addition to the median and interquartile range, mean and standard deviation (SD) were also reported for differences. Statistical analysis was done using IBM SPSS software (USA, version: 20). Standardized mean difference (SMD) were calculated with Glass' Δ approach, an adjusted Hedge's *G*, to indicate the effect sizes using STATA software (version 14). A *p* < .05 is considered significant.

## 3 RESULTS

**FIGURE 1:**
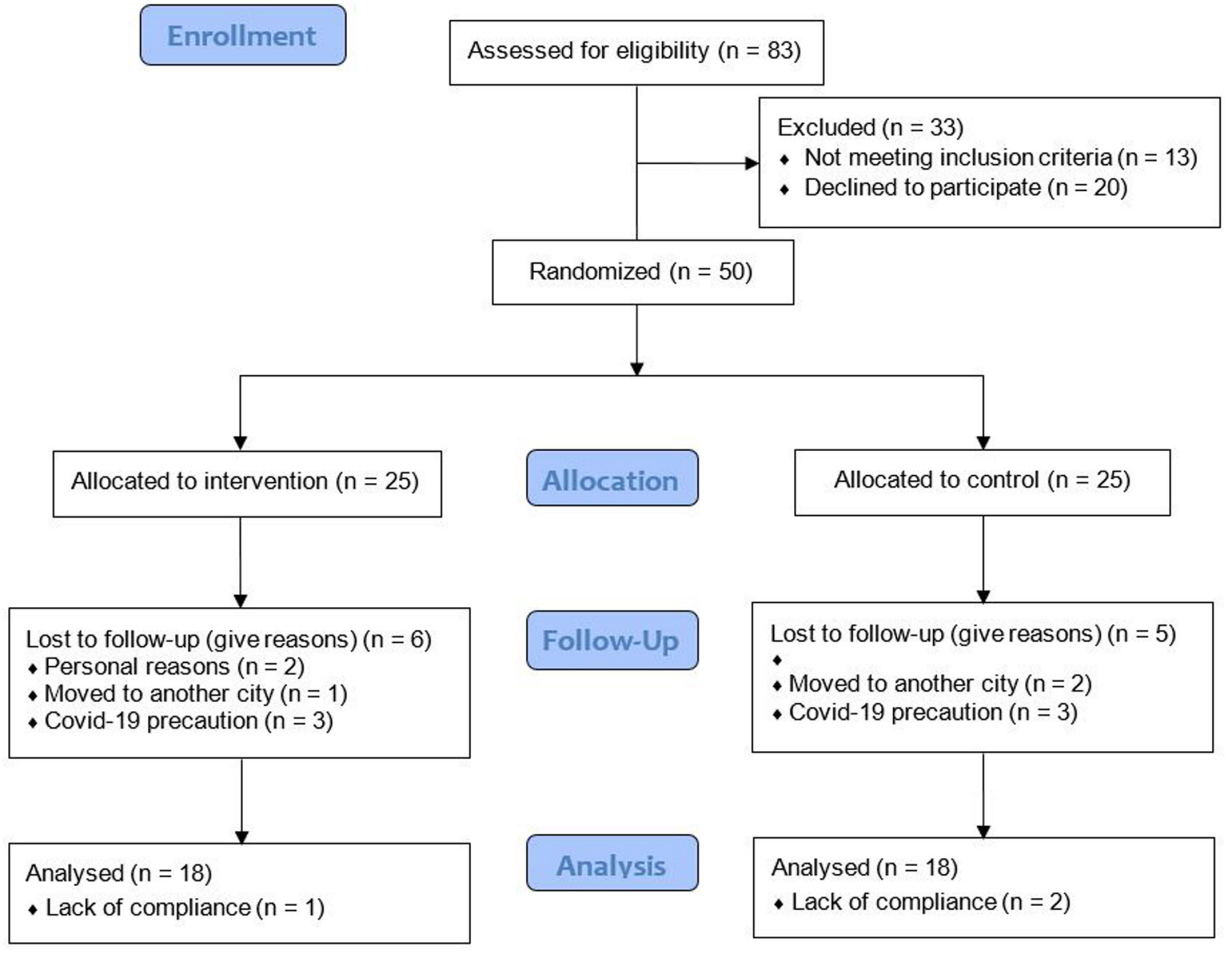
Consort flowchart for participant recruitment and study procedure.

Of patients evaluated for eligibility, 13 were not eligible. Of 70 eligible patients, 50 were willing to enroll, and thus randomly assigned to the study groups. Eleven patients dropped out (personal reasons [*n* = 2], relocated to another city [*n* = 3], not presented for blood test because of COVID‐19 [*n* = 6]). Moreover, three were excluded from statistical analysis due to lack of compliance based on consuming capsules (<80%). Table 1 shows celery powder's chemical and antioxidant properties. No diagnosis of COVID‐19 was reported among participants. In total, in each group, 18 participants (13 males and 23 females) completed the study (Figure 1). Table 2 summarizes the demographic characteristics of the participants. No statistical differences were observed between groups for demographic characteristics of participants. Also, participants reported no side effects according to the consumption of celery powders.

**TABLE 2:** Demographic characteristics of participants.

	Intervention (*n* = 18)	Control (*n* = 18)	*p*
Gender			
Male	7 (38.9)	6 (33.3)	.729^a^
Female	11 (61.1)	12 (66.7)	
Education			
School	14 (77.8)	15 (83.3)	.674^a^
College	4 (22.2)	3 (16.7)	
Marital status			
Single	1 (5.6)	1 (5.6)	1^a^
Married	17 (94.4)	17 (94.4)	
Smoking			
Yes	2 (11.1)	2 (11.1)	1^a^
No	16 (88.9)	16 (88.9)	
Age (year)			
Median (IQR)	56.00 (44.75, 58.25)	56.50 (51.00, 60.00)	.330^b^
Length of T2DM (year)			
Median (IQR)	7.50 (3.50, 12.25)	5.00 (2.50, 12.75)	.763^b^

*Note*: Data are expressed as *N* (%) and median and IQR (25th and 75th percentiles). *p* < .05 considered as significant.

Abbreviations: IQR, interquartile range; SD, standard deviation; T2DM, type 2 diabetes mellitus.

^a^ Chi‐squared test.

^b^ Mann–Whitney *U* test.

### 3.1 Dietary intake and physical activity

Table 3 summarizes dietary intake and physical activity levels for both groups before and after the study. Participants had no significant difference before the intervention for dietary intake and physical activity level. Moreover, during the study period participants did not change their dietary intake for energy (*p* = .528 and .557 for the intervention and control groups, respectively). This is while the reduction in carbohydrate intake was near significant in the intervention group during the study (*p* = .078). Mean changes between groups did not show any significant differences.

**TABLE 3:** Dietary intake and physical activity level of participants, as well as changes during the study.

	Before			After			*p* ^a^	Δ^c^				
	Median	25th	75th	Median	25th	75th		Mean	SD	Median	25th	75th
Energy (kcal)												
Intervention (*n* = 18)	1340.00	1180.75	1793.75	1390.50	1112.25	1543.75	.528	−75.92	601.90	−24.18	−516.83	260.50
Control (*n* = 18)	1356.50	934.30	2049.75	1361.50	907.65	1761.12	.557	684.63	2837.44	−55.66	−695.90	155.92
*p* ^b^	.938			.839				.815				
Carbohydrate (g)												
Intervention (*n* = 18)	232.75	176.37	307.30	210.20	169.07	247.75	.078	−34.18	99.90	−39.30	−100.74	25.97
Control (*n* = 18)	216.25	145.38	299.00	218.10	150.48	294.05	.349	144.09	582.78	−14.57	−92.40	12.93
*p* ^b^	.815			.481				.673				
Protein (g)												
Intervention (*n* = 18)	55.02	37.86	76.60	57.93	43.32	71.57	.647	−1.44	30.19	−2.79	−23.57	17.11
Control (*n* = 18)	57.55	34.55	94.84	48.53	33.36	80.72	.983	26.26	98.06	−1.07	−32.02	24.02
*p* ^b^	.913			.767				.839				
Fat (g)												
Intervention (*n* = 18)	27.10	18.01	35.31	31.78	21.09	39.83	.122	6.12	21.87	14.15	−9.38	22.48
Control (*n* = 18)	30.12	15.67	51.24	23.79	17.94	40.01	.744	4.51	38.78	7.40	−19.33	17.11
*p* ^b^	.673			.628				.426				
Fiber (g)												
Intervention (*n* = 18)	11.04	9.24	20.44	9.68	4.55	14.94	.199	−4.16	11.01	−2.01	−8.57	2.44
Control (*n* = 18)	11.12	7.67	19.31	11.28	8.74	16.16	.679	13.35	90.33	−0.16	−5.66	5.04
*p* ^b^	.839			.389				.767				
Physical activity (MET)												
Intervention (*n* = 18)	33.47	31.87	36.12	33.40	30.95	35.47	.114	1.88	7.09	0.27	−0.41	1
Control (*n* = 18)	34.10	33.06	37.21	34.18	32.60	36.72	.556	0.06	0.83	0.83	−0.48	0.70
*p* ^b^	.226			.203				.501				

*Note*: Data are reported as median and IQR (25th and 75th percentiles) and mean ± SD (standard deviation) for differences. *p* < .05 considered as significant.

Abbreviation: MET, metabolic equivalent.

^a^ Wilcoxon signed rank test.

^b^ Mann–Whitney *U* test.

^c^ Mean difference.

### 3.2 Effect of celery powder on anthropometric indices

Table 4 shows the median and IQR for anthropometrics for groups, before and after the study as well as changes during the study. Anthropometric measurements were not statistically different between groups before the study, except for HC (*p* = .044). The between‐group analysis showed no significant differences in weight after the study (*p* > .05). This is while BMI was reduced in both groups (*p* = .030 and .001 for the intervention and the control groups, respectively).

**TABLE 4:** Anthropometrics measurements of the participants before and after the study, as well as changes during the study.

	Before			After			*p* ^a^	Δ^c^					SMD^d^
	Median	25th	75th	Median	25th	75th		Mean	SD	Median	25th	75th	
Height (m)													
Intervention (*n* = 18)	1.63	1.57	1.73	1.63	1.57	1.73	1	0	0	0	0	0	‐
Control (*n* = 18)	1.62	1.56	1.68	1.62	1.56	1.68	1	0	0	0	0	0	
*p* ^b^	.349			.349				1					
Weight (kg)													
Intervention (*n* = 18)	76.50	71.37	89.65	77.50	67.65	88.25	.031	1.62	3.37	1.50	−1.25	2.15	.264
Control (*n* = 18)	82.00	73.50	86.80	80.50	70.00	85.25	<.001	1.85	1.51	2.00	0.80	3.00	
*p* ^b^	.527			.527				.232					
BMI (kg/m^2^)													
Intervention (*n* = 18)	27.76	25.73	30.12	27.62	25.53	29.52	.030	−0.59	1.18	−0.50	−0.84	0.05	.591
Control (*n* = 18)	30.65	27.33	33.88	30.69	26.62	33.27	.001	−0.73	0.63	−0.71	−1.12	−0.29	
*p* ^b^	.058			.117				.268					
WC (cm)													
Intervention (*n* = 18)	97.00	89.25	99.75	94.50	88.50	100.25	.105	1.05	2.74	0.001	−1.00	2.00	.411
Control (*n* = 18)	102.00	92.75	108.00	99.75	91.62	107.25	.088	1.27	6.34	2.00	−0.62	5.1	
*p* ^b^	.110			.194				.239					
HC (cm)													
Intervention (*n* = 18)	104.00	98.75	112.25	103.50	97.37	107.75	.034	2.13	5.42	1.50	0.37	3.12	.369
Control (*n* = 18)	113.50	101.75	120.50	105.75	99.75	115.62	.004	4.44	7.85	2.50	0.75	5.00	
*p* ^b^	.044			.189				.325					
BF (%)													
Intervention (*n* = 18)	33.05	24.90	41.22	32.75	23.60	39.62	.021	1.38	2.88	0.95	−0.05	2.00	.482
Control (*n* = 18)	36.45	25.60	41.97	38.10	25.97	41.22	.513	−1.35	10.16	0.60	−1.30	1.95	
*p* ^b^	.591			.271				.326					

*Note*: Data are reported as median and IQR (25th and 75th percentiles) and mean ± SD (standard deviation) for differences. *p* < .05 considered as significant.

Abbreviations: BF, body fat; BMI, body mass index; HC, hip circumference; SMD, standardized mean difference; WC, waist circumference.

^a^ Wilcoxon signed rank test.

^b^ Mann–Whitney *U* test.

^c^ Mean difference.

^d^ Glass' Δ.

### 3.3 Effect of celery powder on glycemic indices and lipid profile

Table 5 describes the glycemic indices and lipid profile of participants during the study. Participants had no differences in glycemic indices before the study. FBS was slightly diminished in both groups, but the reductions were not significant. The within‐group and differences between groups did not show any beneficial effect of the intervention on glycemic indices. Insulin was increased in the intervention group (1.41 ± 4.81 mU/mL), while it was reduced in the control group (−2.32 ± 10.10 mU/mL). Although the result for insulin levels was not statistically different, the trend of changes was clinically valuable.

**TABLE 5:** Cardiometabolic indices of the participants before and after the study, as well as changes during the study.

	Before			After			*p* ^a^	Δ^c^					SMD^d^
	Median	25th	75th	Median	25th	75th		Mean	SD	Median	25th	75th	
FBS (mg/dL)													
Intervention (*n* = 18)	148.50	130.00	191.50	157.50	129.50	191.25	.632	−2.61	32.68	−1.00	−28.75	15.25	.024
Control (*n* = 18)	172.00	140.25	200.75	172.00	135.25	198.75	.879	−2.16	57.81	7.00	−24.25	20.75	
*p* ^b^	.496			.669				.601					
Insulin (μIU/mL)													
Intervention (*n* = 18)	10.03	8.76	17.07	10.64	6.27	16.26	.267	1.41	4.81	1.79	−2.1	3.79	.418
Control (*n* = 18)	12.63	9.76	19.02	14.93	7.60	20.78	.500	−2.32	10.10	−0.12	−5.26	3.22	
*p* ^b^	.506			.184				.206					
HOMA‐IR													
Intervention (*n* = 18)	4.70	3.21	6.09	4.74	2.35	7.08	.586	0.49	3.40	0.68	−1.87	1.25	.381
Control (*n* = 18)	5.46	3.51	7.21	5.68	3.01	9.70	.948	−1.08	5.97	0.54	−4.09	2.24	
*p* ^b^	.467			.327				.899					
HOMA‐B													
Intervention (*n* = 18)	55.24	30.51	70.66	40.58	24.12	49.49	.085	6.73	25.48	8.00	−2.36	20.10	.394
Control (*n* = 18)	41.89	27.68	66.20	52.55	19.98	106.42	.845	−4.50	42.80	2.37	−36.88	13.58	
*p* ^b^	.569			.376				.343					
TG (mg/dL)													
Intervention (*n* = 18)	147.00	127.50	243.50	188.50	125.25	247.00	.695	−2.33	76.79	7.50	−41.50	19.75	−.204
Control (*n* = 18)	155.00	97.50	226.00	145.50	122.00	214.00	.296	−15.77	80.14	−8.50	−47.50	25.75	
*p* ^b^	.429			.359				.624					
Cholesterol (mg/dL)													
Intervention (*n* = 18)	169.50	152.75	219.50	184.00	166.00	204.75	.368	−4.16	22.90	−2.50	−25.25	8.75	−.378
Control (*n* = 18)	161.00	135.75	183.25	160.50	129.00	185.00	.663	−4.94	41.82	−4.50	−37.50	15.75	
*p* ^b^	.169			.094				.950					
HDL‐c (mg/dL)													
Intervention (*n* = 18)	44.50	35.00	54.00	42.00	36.00	48.50	.377	2.22	7.20	0.001	−1.75	5.50	.228
Control (*n* = 18)	42.50	33.00	49.00	45.00	32.75	52.00	.393	−1.83	8.94	−1.00	−4.00	2.00	
*p* ^b^	.704			.763				.267					
LDL‐c (mg/dL)													
Intervention (*n* = 18)	89.50	81.25	125.25	104.00	87.25	124.00	.616	−3.88	18.52	−0.50	−14.25	11.50	−.466
Control (*n* = 18)	85.00	67.00	103.50	85.50	58.25	103.75	.760	−2.55	28.92	0.001	−27.25	13.00	
*p* ^b^	.194			.054				.987					
Systolic BP (mmHg)													
Intervention (*n* = 18)	135.00	125.00	147.00	130.00	119.50	139.25	.011	6.50	10.13	3.50	1.00	17.25	.156
Control (*n* = 18)	140.00	128.25	153.75	128.00	121.00	144.25	.009	8.94	11.98	10.50	0.50	18.25	
*p* ^b^	.318			.812				.334					
Diastolic BP (mmHg)													
Intervention (*n* = 18)	88.00	80.75	90.50	80.00	77.50	85.50	.002	4.72	4.93	4.50	1.50	8.00	.422
Control (*n* = 18)	88.50	86.00	91.50	85.50	80.75	88.50	.015	4.38	11.55	5.00	1.50	10.25	
*p* ^b^	.205			.078				.751					
AST (IU/L)													
Intervention (*n* = 18)	20.00	16.00	27.00	18.00	15.25	22.25	.106	13.83	44.09	2.50	−2.00	8.00	.283
Control (*n* = 18)	19.50	17.00	24.00	19.50	16.00	24.00	.909	−0.66	6.46	0.001	−2.50	3.25	
*p* ^b^	1.000			.515				.234					
ALT (IU/L)													
Intervention (*n* = 18)	24.50	18.00	32.25	22.50	17.50	27.50	.111	3.61	8.21	2.00	−2.25	8.00	.251
Control (*n* = 18)	24.50	19.75	28.00	26.00	17.75	33.50	.979	0.05	8.83	0.001	−6.25	−0.05	
*p* ^b^	.886			.590				.267					
MDA (μmol/L)													
Intervent on (*n* = 18)	1.93	1.79	2.16	2.10	1.58	2.23	.407	−0.05	0.26	0.001	−0.20	0.10	−.719
Control (*n* = 18)	1.84	1.65	2.05	1.78	1.53	2.00	.061	0.18	0.37	0.12	−0.05	0.31	
*p* ^b^	.401			.044				.062					

*Note*: Data are reported as median and IQR (25th and 75th percentiles) and mean ± SD (standard deviation) for differences. *p* < .05 considered as significant.

Abbreviations: ALT, alanine transaminase; AST, aspartate transaminase; BP, blood pressure; FBS, fasting blood sugar; HOMA‐IR, homeostatic assessment model for insulin resistance; HOMA‐B, homeostatic assessment model for β‐cell function; HDL, high‐density lipoprotein; LDL, low‐density lipoprotein; MDA, malondialdehyde; SMD, standardized mean difference; TG, triacylglycerol.

^a^ Wilcoxon signed rank test.

^b^ Mann–Whitney *U* test.

^c^ Mean difference.

^d^ Glass' Δ.

Baseline lipid profile levels between groups were not significantly different. Changes during the study for lipid profile were not significant. Moreover, the changes in blood lipids between placebo and celery powder groups did not show any statistical differences (*p* > .05).

### 3.4 Effect of celery powder on blood pressure, ALT, AST, and MDA

Blood pressure was not statistically different between groups before the study (*p* > .05). Analysis showed significant increments in both systolic (*p* = .011 and .009 for the intervention and control groups, respectively) and diastolic (*p* = .002 and .015 for the intervention and control groups, respectively) blood pressure within groups. But changes were not significantly different between groups (*p* = .334 and .751 for the systolic and diastolic blood pressure, respectively). Analysis for ALT and AST did not reveal any significant differences. Table 5 showed results for blood pressure, ALT, AST, and MDA. MDA showed a slightly significant difference between groups after the study (*p* = .044), also, the changes for MDA were not statistically different between groups (*p* = .062).

## 4 DISCUSSION

To the best of our knowledge, no high‐quality investigation has assessed the effect of celery on cardiometabolic factors in overweight/obese individuals with T2DM. Thus, in this study, we pilot the effect of celery powder on the anthropometrics, biochemical, and blood pressure of individuals with T2DM with exceeded weight. Our study did not show any significant effects of celery powder on the anthropometrics, fasting blood glucose, metabolic, and blood pressure. Although changes were not statistically significant, insulin and MDA levels showed a clinical improvement in the intervention group.

Dietary fiber intake, independent of energy restriction, can improve weight status, BMI, and waist circumference (Chen et al., 2018; Jovanovski et al., 2020). In our study, participants did not significantly change their fiber consumption through their diet. The analysis of celery powder in our study showed the existence of 10.51 g of fiber in 100 g of the powder. A previous meta‐analysis showed that 16 g of dietary fiber can cause weight loss (Thompson et al., 2017). Thus, based on the amount of fiber content of celery powder and the dosage used in this trial, the increased fiber content cannot lead to the reduction of anthropometric indices. There are some possible reasons that explain why we did not find any significant effects after celery consumption. Low sample size, short duration, and low dosage can be the main factors. In addition, the present investigation was done during the COVID‐19 outbreak, which might affect our results through lower physical activity among participants. Also, the adherence to the hypocaloric dietary plan during the trial was low. These can confound the possible effect of celery supplementation on the cardiometabolic factors.

Dietary fibers are suggested to improve anthropometric indices by means of various pathways including a reduction in energy intake, absorption rate of macronutrients, and appetite suppression (Chen et al., 2018). Gut microbiota amendment is also suggested for dietary fiber in weight reduction (Wang et al., 2021).

Celery has been shown to reduce preprandial blood glucose in prediabetic adults (Yusni et al., 2018). In the study of Yusni et al. (2018), participants received 750 mg/day of celery extract. The discrepancy seen between this study and our work could be due to the intervention used [celery extract vs. powder in Yusni et al.'s (2018) study and ours, respectively]. The antihyperglycemic effect of celery has been linked to the presence of various compounds such as luteolin and apigenin (Hedayati et al., 2019). Luteolin can suppress cytokine release in signaling pathways that are dependent on nuclear factor kappa B (NF‐κB) (Kim et al., 2014). On the other hand, apigenin has the properties to decrease hepatic glucose‐6‐phosphatase, lower serum glucose, and increase the concentration of insulin (Panda & Kar, 2007). These can support our findings. Although our results were not significant, the insulin level increased in our intervention group, while in the control group insulin was reduced. In addition, in both groups, the fasting blood glucose was decreased, but the reduction was higher in the intervention group. This insignificant result could be due to the low dosage and the low duration of the study.

In our study, TG, total cholesterol, and LDL‐c insignificantly lowered in both groups. It should be noted that, after the intervention, HDL‐c increased in the intervention group and decreased in the control group nonsignificantly. As 20% of total sugar in celery is 1,3‐beta‐glucan, this may justify its lipid‐lowering effects (Ko & Lin, 2004). Also, according to research on hypercholesterolemic patients, beta‐glucan‐rich oat bread reduced blood cholesterol (Momenizadeh et al., 2014). Animal model intervention with celery leaf was shown to lower lipid profile components including TG, total cholesterol, LDL, and increase HDL levels (Dianat et al., 2015). Based on the results of a previously conducted meta‐analysis, beta‐glucan can lower total cholesterol and LDL‐c, but it had no effects on TG and HDL‐c (Zhu et al., 2015). As mixed results have been seen, the hypolipidemic effect of celery could not be due to the presence of beta‐glucan alone. The observed changes in lipid profile in our study can be supported by a research done by Dianat et al. (2015) in which hydroalcoholic celery leaf extract was used. Thus, it can be interpreted that the lipid‐lowering effect of celery could majorly be due to its active phytochemical compounds and not fiber content.

An active compound of celery called *A. graveolens* exerts hypotensive effects by the antagonistic activity of Ca^2+^ channels (Jorge et al., 2013). Also, luteolin in celery leaf induces a γ‐aminobutyric acid (GABA)‐induced antihypertensive effect (Dianat et al., 2015). Dewi et al. (2010) showed the celery ethanol extract was able to reduce both systolic and diastolic blood pressure. We did not see the same results in our study. Various possible reasons can justify these variations in the results. First, participants in this study were overweight or obese, which can affect blood pressure, and the median of recorded blood pressure showed an increased blood pressure among our participants, but they were not suffering from hypertension. This is while the baseline assessments of blood pressure in Dewi et al.'s (2010) study indicated participants are normotensive male adults. These mixed results could also be due to the different forms of used celery (powder vs. extract). Shayani Rad et al. (2022) observed blood pressure reduction effect of celery seed extract. There are some differences in their study design in comparison with ours which can justify the discrepancies. Although the abovementioned study had shorter duration, they administered seed extract with higher dosage which can show different health promoting effect. Furthermore, individuals recruited in Shayani Rad's study were hypertensive patients, while in our study the participants were normotensive. Thus, this might affect our observations in comparison with Shayani Rad's study.

Ingestion of potassium both through food or supplementation can lower blood pressure, especially when adequate intake of potassium (3500 mg/day or 90 mmol/day) is achieved (Filippini et al., 2017). Thus, based on the high potassium content of celery, it can help to ameliorate blood pressure. In addition to potassium, celery contains magnesium, which show magnesium supplementation in patients with prediabetes can lower blood pressure (Dibaba et al., 2017). But it should be noted that the dosage for supplementation used in the trials included in the abovementioned meta‐analysis (Dibaba et al., 2017; Filippini et al., 2017) were higher than ours (25–250 mmol/day for potassium and 365–450 mg/day magnesium in meta‐analysis vs. 20 mg/day and 2.3 mg/day for potassium and magnesium, respectively, in our study).

In this trial, no significant changes were seen in serum MDA levels. It was shown that higher dietary antioxidant capacity is related to lower serum levels of MDA (Abshirini et al., 2019). In the natural history of T2DM, elevated levels of lipid peroxidation occur before further complications of diabetes (Soliman, 2008). MDA is a stable final product in the pathway of lipid peroxidation (Nielsen et al., 1997), which can lead to vascular complications and increase the risk of mortality (Mishra & Mishra, 2017). Thus, the reduction of MDA in our trial after celery consumption could be due to the antioxidant substances of celery including vitamin C, beta‐carotene (provitamin A), and manganese (Hedayati et al., 2019), but, as we used a low dosage, these changes were insignificant.

As no high‐quality investigation has assessed the effects of the celery‐related intervention on human subjects, estimation of a minimum required sample size and selecting a proper dose for intervention was not possible. Thus, we had to run a pilot study in order to prevent the loss of resources. Therefore, the limited number of participants and a low dose of celery used were the main potential barriers in the present investigation. Further studies are recommended with higher sample size, longer duration, proper dose, and type of substances including alcoholic extract for intervention. Moreover, in the near future, it would be interesting to conduct a new experiment with apparently healthy people, with the intention of observing the potency of this natural product. It should be noted that various outcomes evaluated in this study may help other investigators to conduct further high‐quality projects to find possible beneficial effects of celery. Meanwhile, conducting this intervention during the COVID‐19 outbreak imposed some problems on our study including a lack of adherence to recommended diet, and lowered physical activity levels due to lockdowns which were inevitable. Also, the number of missing patients was relatively high (about 25%) with reasons of moving to another city or not presenting at the laboratory for the second blood test, which both are stated to be due to the COVID‐19 outbreak. Measuring other oxidative stress and inflammatory markers such as C‐reactive protein (CRP), interleukin‐6 (IL‐6), tumor necrosis factor alpha (TNF‐α), and vascular indices are suggested for future studies. Moreover, determining the exact amount of major antioxidants in celery could fill the gap regarding the potential involved mechanisms of this herb.

## 5 CONCLUSION

Celery powder did not improve cardiometabolic indices during the 12‐week intervention. Further studies with a higher sample size and dosage and longer duration are recommended.

## AUTHOR CONTRIBUTIONS

**Mohammad Ali Mohsenpour:** Conceptualization (equal); data curation (equal); formal analysis (equal); investigation (equal); methodology (equal); writing – original draft (equal); writing – review and editing (equal). **Mahsa Samadani:** Data curation (equal); investigation (equal); writing – original draft (equal). **Zeinab Shahsavani:** Data curation (equal); investigation (equal); writing – original draft (equal). **Mohammad‐Taghi Golmakani:** Conceptualization (equal); formal analysis (equal); investigation (equal); methodology (equal); writing – original draft (equal). **Gholam Reza Pishdad:** Conceptualization (equal); data curation (equal); investigation (equal); writing – original draft (equal). **Maryam Ekramzadeh:** Conceptualization (equal); data curation (equal); formal analysis (equal); funding acquisition (equal); investigation (equal); methodology (equal); project administration (equal); supervision (equal); writing – original draft (equal); writing – review and editing (equal).

## FUNDING INFORMATION

The vice chancellor of research of the Shiraz University of Medical Sciences financially supported this study (code: 20145).

## CONFLICT OF INTEREST STATEMENT

The authors declare that they do not have any conflict of interest.

## ETHICS STATEMENT

All procedures performed in studies involving human participants were in accordance with the Declaration of Helsinki and study protocol was approved by the Ethics Committee of Shiraz University of Medical Sciences (reference ID: IR.SUMS.REC.1398.1381). The study was registered retrospectively in the Iranian Registry of Clinical Trials (IRCT, www.irct.ir) under the registration ID: IRCT20100223003408N8. Before including in the study, participants were informed about the study and their rights, then a written informed consent was obtained from participants, but they were free to choose whether to continue the study or not. All the assessments and laboratory tests were free of charge.

## Data Availability

The datasets used and/or analyzed during the current study are available from the corresponding author on reasonable request.
